# Epigenome comparisons reveal linkage between gene expression and postnatal remodeling of chromatin domain topology

**DOI:** 10.1371/journal.pone.0191033

**Published:** 2018-02-21

**Authors:** Bruce H. Howard, Tazuko H. Hirai, Valya R. Russanova

**Affiliations:** Division of Developmental Biology, Eunice Kennedy Shriver National Institute of Child Health and Human Development, National Institutes of Health, Bethesda, Maryland, United States of America; Pohang University of Science and Technology, REPUBLIC OF KOREA

## Abstract

Substantial evidence has accumulated linking epigenome change to alterations in stem cell function during postnatal development and aging. Yet much remains to be learned about causal relationships, and large gaps remain in our understanding of epigenome-transcriptome interactions. Here we investigate structural features of large histone H3K27me3-enriched regions in human stem cell-like monocytes and their dendritic cell derivatives, where the H3K27me3 modification is considered to demarcate Polycomb (PcG) domains. Both differentiation- and postnatal development-related change are explored, initially by confirming expected reciprocal relationships between transcript abundance and span of PcG domains overlapping transcribed regions. PcG-associated postnatal transcriptome change specific to the stem cell-like monocytes is found to be incompletely explained by conventional measures of PcG region structure. To address this, we introduce algorithms that quantify local nucleosome-scale conservation of PcG-region topology. It is shown that topology-based comparisons can reveal broad statistical linkage between postnatal gene down-regulation and epigenome remodeling; further, such comparisons provide access to a previously unexplored dimension of epigenome architecture.

## Introduction

Over the past several years, multiple studies have appeared linking epigenome structure to shifts in stem cell differentiation patterns [1], cell senescence [2], and lifespan determination [3–6]. With respect to longevity, rapid progress has been driven in large part by model systems, where manipulation of epigenetic control pathways, including those responsible for the balance between histone acetylation or histone methylation, can have substantial impacts. Areas of uncertainty persist, since interventions to test the role(s) of epigenome function on longevity do not always yield consistent results in different organisms [7]. Further, it remains a challenge to establish mechanisms by which genetic manipulations influence longevity, e.g., by broad perturbation of epigenome structure vs. altered expression of selected growth- or differentiation-control genes [4]; and much remains to be done in mapping epigenome structures of model systems onto age-related transcriptome change.

An approach complementary to work in model systems is to characterize epigenome structure through lifespan in vertebrate organisms, especially humans. Along these lines, there is widespread interest in using epigenetic clocks [8–11], in particular those based on machine learning-defined DNA methylation profiles [12, 13]. Such clocks serve both as aging biomarkers and to evaluate relationships between postnatal development and aging [14]. While causality has not been addressed directly in early studies, the manipulation of short-lived vertebrate model systems in conjunction with use of these clocks may be informative in the future. For the most part, strong linkage has not been reported between age-related change in DNA methylation-based patterns and transcriptome function [3, 15–17].

With respect to the chromatin-based epigenome, substantial structural alterations have been found to accompany cellular senescence [2, 18, 19]. Senescence pathways exhibit evolutionary conservation, and since the accumulation of senescent cells in vertebrate tissues can compromise tissue function, this should continue to be a productive area of investigation. On a cautionary note, transcriptome and epigenome changes through lifespan appear to be much more variable than, and in general distinguishable from, those observed in senescence pathways.

Stem cell malfunction is considered to be a fundamental feature of the aging phenotype [1, 20, 21], thus how stem cell functions evolve through lifespan merits much further attention. Mouse whole genome studies have delineated age-related chromatin change in hematopoietic stem cells (HSC)[16] and in muscle stem cells [22]. In HSC, broadening of histone H3K4me3 peaks was observed, while both studies reported increases in repressive histone H3K27me3 marks. H3K4me3 varied positively with age-related gene expression in HSC, whereas statistically significant inverse correlations between H3K27me3 change and gene expression appeared to be limited or absent. Notably, in these and other non-stem cell studies, H3K4me3 and H3K27me3 marks were quantified over fixed regions flanking transcription start sites, i.e., without specific attention to identification of chromatin domain boundaries.

Efforts to map long-range regulatory interactions in the context of development have largely focused largely on 3C-based approaches [23, 24]. Topologically associated domains (TAD) and sub-domains have been observed to remodel in embryonic stem cells [24], although these cells may have considerably greater plasticity in terms of 3D architecture than postnatal lineages. DNase hypersensitivity site-based studies [25] and super-resolution microscopy [26] provide complementary technologies to shed light on development-linked chromatin structure changes. Nuclear architecture is also dependent on the proper function of lamins; thus disorders of these proteins are of interest [27], as are reported lamin-Polycomb (PcG) protein interactions [28].

Here we investigate the structure and function of H3K27me3-enriched (PcG) domains that overlap gene bodies in human monocytes and dendritic cells. From the analysis of RNA-seq data, taken together with the evaluation of differentiation- and age-associated remodeling of such domains, results emerge which point to local shifts in conservation of nucleosome-scale topology as an important independent measure of epigenome change and its consequences on transcriptome function.

## Results

### Identification of H3K27me3-enriched (PcG) domains

**Fig 1:**
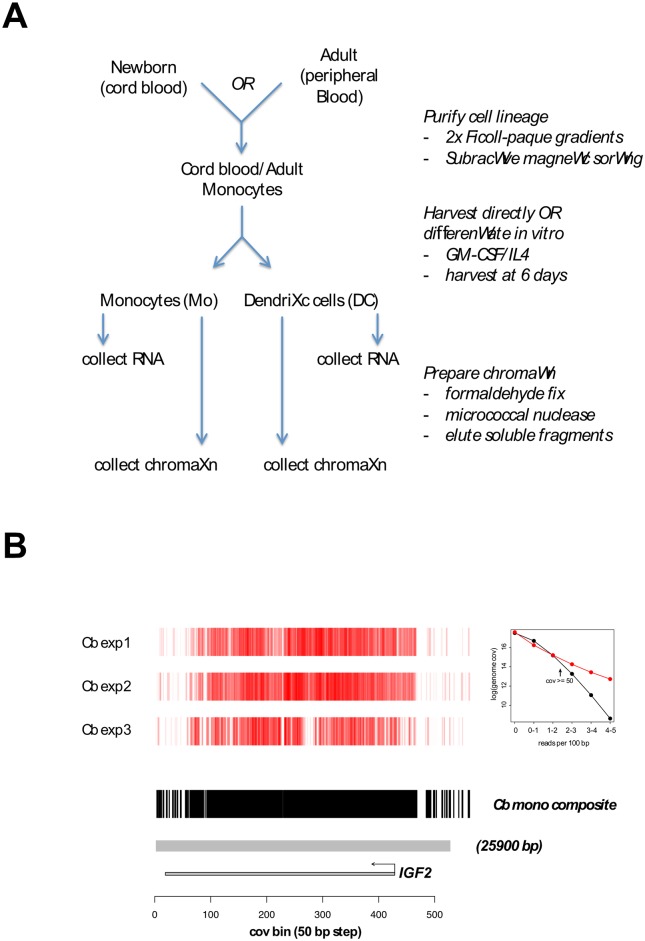
Experimental design and region identification. A. Schematic of experimental flow. B. H3K27me3-enriched region overlapping the IGF2 gene. Heat map depictions of H3K27me3 (PcG)-enrichment are shown for several independent experiments. Algorithms to estimate composite enrichment (threshold exceeded in a specified fraction, here at least 2 of 3, of experiments), apply filters, and set region boundaries (shown by gray bar) are described in Methods. Inset. Threshold for coverage enrichment. Based on a minimal 100 bp summation unit (50 bp steps) and 36 bp reads, coverage sums greater than the specified threshold were totaled genome-wide (y-axis). Red points represent observed profile for newborn monocytes, whereas black points show values predicted for a Poisson distribution.

In order to investigate links between chromatin domain structure and expression of genes embedded in those domains, we performed experiments comparing samples derived from paired differentiation states (monocytes and dendritic cells), as well as from different lifespan stages (newborn, young adult and old adult). The experimental design is summarized in Fig 1A.

While RNA analysis in this study followed standard methods, we opted in the analysis of chromatin domains to explore new approaches. Repressive regions on a scale of kilobases to megabases are thought to be functionally important in the spatial organization of metazoan genomes [26]. At the same time, compelling evidence exists that nucleosome- and enhancer-scale regulatory domains, typically hundreds of bases in size, are key determinants of differentiation and developmental states [25]. Given these considerations, we developed algorithms that define boundaries of relatively large (median 10 kb) regions of H3K27me3 enrichment while permitting nucleosome-scale stochastic variation and small internal gaps. In these algorithms, enrichment is scored by where experimental coverage (red, inset in Fig 1B) exceeds that predicted by a Poisson distribution (black). Depicted in the main Fig 1B are heat maps and a composite representation used to delineate a 26 kb region spanning the IGF2 gene in cord blood (Cb) monocytes. This region is conserved in adult (young and old) monocytes (S1 Fig). Of twelve regions with the greatest span (range 82–178 kb), four overlapped the HOXA, HOXB, HOXC, or HOXD gene clusters (S1 Table). Other domains in this group included the PCDHG cluster-associated PcG region (119 Kb), which was reported previously to exhibit postnatal DNA methylation change [15], as well regions overlapping the DOCK9 and SYT2 genes. Regulation of the latter, it has been proposed, is based in part on DNA methylation-based epigenetic memory [29, 30].

### Large PcG-enriched domains associated with strongly repressed gene expression

**Fig 2:**
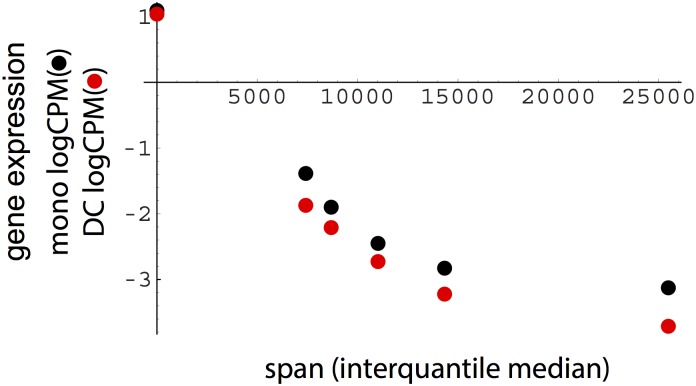
Association of PcG region span with reduced RNA levels. For both monocytes and dendritic cells (DC), expression (RNA-seq log counts per million) exhibited a strong negative correlation with the span of overlapping regions. Points designate no PcG region overlap (y-axis), or median span values (from left to right, quantile ranges: 0–0.25, 0–0.5, 0.25–0.75, 0.5–1, 0.75–1). Monocyte comparison for quantiles 0–0.25 vs. 0.75–1, p < 6.9 10^−12^, Wilcox test. DC comparison for quantiles 0–0.25 vs. 0.75–1, p < 3.1 10^−14^, Wilcox test.

A key goal of this study was to relate H3K27me3-enriched chromatin domain structure to gene expression. As a first step, RNA-seq data were analyzed from cord blood and adult sources to determine whether transcribed regions that overlap H3K27me3 domains exhibit lower mean expression levels. This was clearly evident, although the span of repressive regions strongly influenced the negative correlation between overlap and expression (Fig 2; p < 6.9 10^−12^ for monocytes; p < 3.1 10^−14^ for DC). Near maximal down-regulation in mean expression (>50-fold in both monocyte and dendritic cell data sets) is not reached until median region lengths approach 15 kb (Fig 2). On the basis of super-resolution microscopy, PcG repressive domains have been reported to exhibit distinct scaling properties such that packing density increases with increased domain length [26]; thus, insofar as higher packing density results in greater repression of gene expression, our results support and extend current imaging-based evidence.

### Differentiation-linked gene expression vs. PcG region span

**Fig 3:**
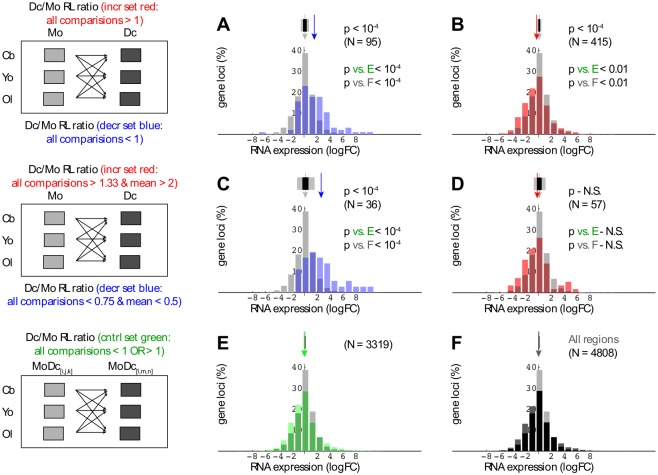
Inverse relationships between region span and differentiation-dependent gene expression change. Cord blood RNA-seq log fold change (logFC) values are presented as overlapping histograms, with explanatory schematics on left for rows AB, CD, and EF. For rows AB and CD, RL (region length) ratios were compared according to two criteria: i) all nine Mo/Dc RL ratios either exceeded the indicated ‘all comparisons’ filter threshold (increase set shown in red) or fell below the designated threshold (decrease set shown in blue), and ii) the mean of the same nine Mo/Dc RL ratios either exceeded or fell below the corresponding mean threshold value. In panel F, the black histogram shows values for genes where any part of the transcribed region overlapped at least one H3K27me3-enriched region. In each panel, the reference light gray histogram represents all gene loci (N = 23710) for which the data yielded measurable expression (min logCPM = -4.67). Arrows above histograms indicate median reference and subset expression values, while flanking (or overlapping) black and gray rectangles correspond to random sampling-derived p values of 0.005 and 10^−4^, respectively.

Cord blood and adult peripheral blood monocytes retain stem cell-like properties, and can be induced to differentiate into dendritic cells (DCs) in vitro with an efficiency that is essentially complete [31]. We exploited the latter property to optimize and validate algorithms relating gene expression to change in H3K27me3 region size. Keeping in mind that substantial stretches of the epigenome exhibit high variability, differentiation-linked chromatin change was scored as occurring only if consistent for all of nine comparisons (schematics on left, Fig 3), where each monocyte or dendritic cell span estimate reflected three independent ChIP-seq experiments. RNA-seq results (32 datasets in total) were derived from cord blood and adult peripheral blood monocytes, as well as the corresponding in vitro differentiated dendritic cells.

On examination of the data, the strongest differentiation-associated link between PcG region and gene expression change was up-regulation in median RNA level coupled to decreased domain size. This inverse relationship was reproducible in being observed with independent cord blood (Fig 3) and adult RNA (S2 Fig) datasets, and was significant by 10K random sampling with replacement (p < 10^−4^). Statistical significance was comparable for genes exhibiting inverse expression-domain size change relative to: i) all 23710 expressed genes in the analysis (Fig 3A and 3C, S2AC Fig, ii) genes overlapping any conserved PcG domain (see Fig 3F, S2F Fig), or iii) genes overlapping domains with comparable size variation not linked to differentiation, i.e., based not on monocyte-DC comparisons, but rather on pairs derived by permutations of monocyte-DC datasets (see Fig 3E, S2E Fig).

**Fig 4:**
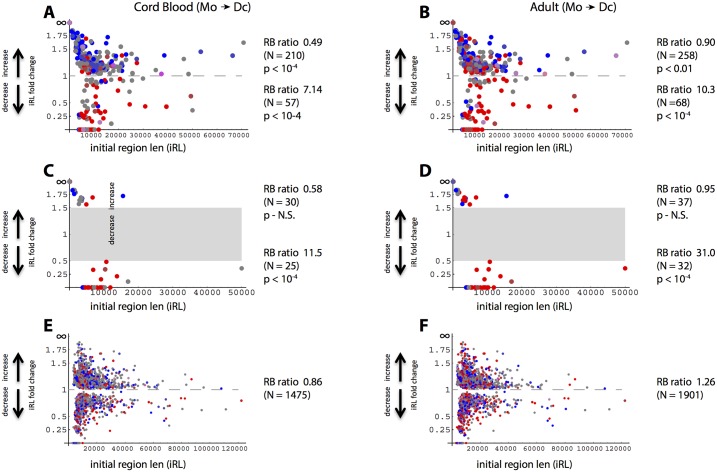
Region span vs. significant differentiation-dependent gene expression change. PcG regions, plotted by initial span (x-axis) and monocyte to DC differentiation-linked fold change (y-axis), were assigned colors according to the ratio of overlapped genes that exhibit significant (FDR < 0.05) change in expression. Points representing regions with significantly increased RNA levels are red, decreased levels are blue, whereas regions overlapping equal numbers of up- and down-regulated genes are gray. Intermediate hues reflect either: i) proportionality, if the most but not all of the genes associated with a given region exhibited consistent significant expression change, or ii) multiple regions associated with different predominant gene expression patterns and represented by points that overlap. Panels A and B show only regions where the differentiation-linked fold change in span was consistent, i.e, > 1 in all nine monocyte-DC comparisons or < 1 in all comparisons. Panels C and D show only regions where the differentiation-linked fold change in span was consistent and either exceeded or fell below threshold values. Gray rectangles in panels CD denote areas where the threshold filter excluded regions from being plotted. Random sampling with replacement was used to derive p-values for RB (red-blue) ratios, where regions overlapping all or a majority of significantly up- or down-regulated genes were pooled.

We further evaluated the inverse expression-domain size relationship for the subset of genes whose RNA levels changed significantly (FDR (Benjamini-Hochberg) < 0.05) in either cord blood or adult datasets. In Fig 4, the direction of expression change is depicted by color so as to permit plotting of initial H3K27me3-enriched region length (iRL) vs. length change. Here again expression up-regulation was statistically linked to decreased domain size (Fig 4A, 4B, 4C and 4D, p < 10^−4^ by 10 K random sampling) for both the independent cord blood and adult RNA datasets. No association was observed for overlap with any PcG-enriched region (Fig 4F) or with regions selected by permutations of monocyte-DC datasets (Fig 4E). Potentially of interest, the largest PcG region (50 kb point in Fig 4C and 4D) showing a >1.5-fold differentiation-linked decrease in length was the lamin A (LMNA) gene (see S3A Fig). This case is atypical, however, in that LMNA RNA levels are exceptionally high for association with such a large PcG domain (S3B Fig), and that only a small decrease in mean H3K27me3-enrichment is evident for the adult samples (S3D Fig).

### Postnatal gene expression vs. PcG region size

**Fig 5:**
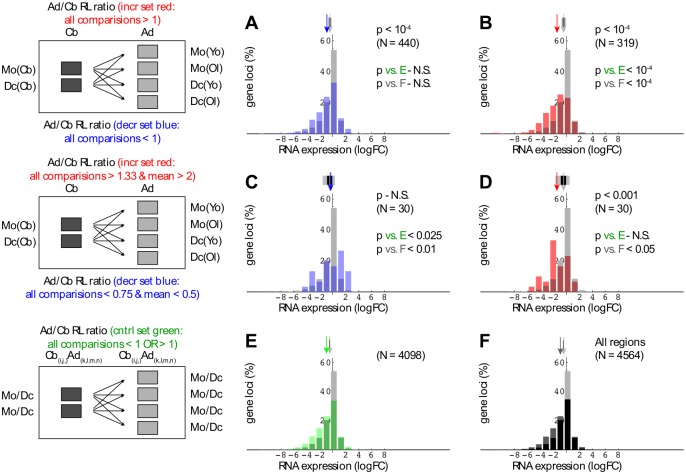
Associations between region span and postnatal monocyte gene expression change. Candidate change regions were identified based on consistent cord blood-adult span differences in multiple comparisons (explanatory schematics on left). While region estimates are derived from both monocyte and DC data, only monocyte RNA-seq data are shown in this figure (for DC expression data, see Fig 6). Minimum monocyte logCPM = -4.21. Plotting conventions are as in Fig 3.

**Fig 6:**
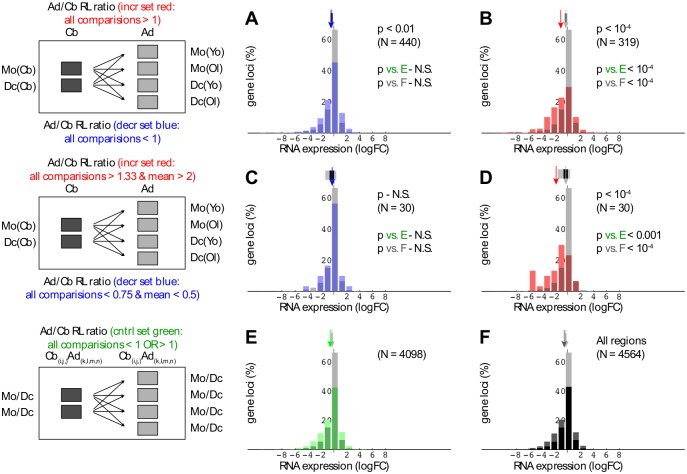
Associations between region span and postnatal dendritic cell gene expression change. Analysis identical to that in Fig 5, except dendritic cell (DC) RNA-seq data (minimum logCPM = -4.62) are shown. Plotting conventions are as in Fig 3.

**Fig 7:**
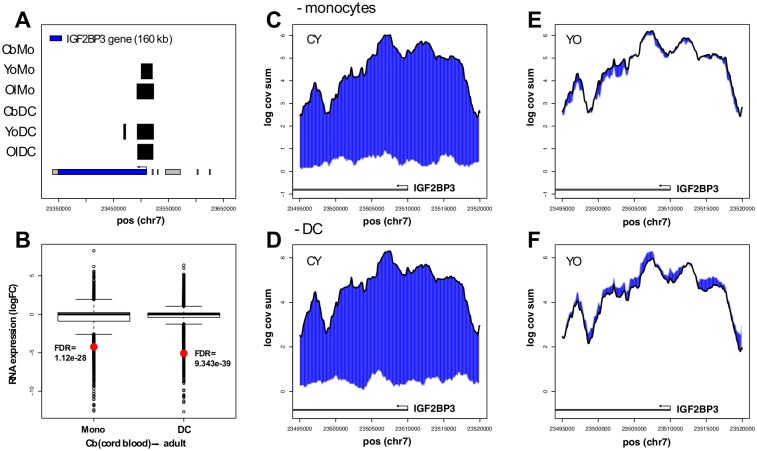
IGF2BP3-associated PcG region: Newborn vs. adult profiles. A. Estimated region boundaries for a relatively large (approx. 27 kb) H3K27me3-enriched domain overlapping the IGF2BP3 promoter region in adult samples. B. Postnatal down-regulation of IGF2BP3 monocyte and DC expression (log fold change). Panels CDEF represent coverage profile comparisons for this region (2 kb resolution, 50 bp steps), with differences highlighted in blue. CY, cord blood-young; YO, young adult-old adult. The gray and black lines that form the boundaries of the highlighted blue region denote cord blood (panels CD) or young (panels EF), and young (panels CD) or old (panels EF), respectively. The gray bars with arrows at bottom indicate the transcribed IGF2BP3 region that falls within the plotted region.

To explore interrelationships between postnatal development, PcG region structure, and gene expression, we applied a variant of the above analysis to comparisons between cord blood and adult samples. Interpretation is limited in view of the cross-sectional design of this part of the study; nevertheless, with notable differences, PcG region change appears to occur on a scale comparable to that seen above. In contrast to differentiation, a negative correlation between PcG region and postnatal expression change is only observed for down-regulation in median RNA level in association with increased domain size (Figs 5B, 5D and 6B and 6D, representing monocyte and DC RNA-seq data, respectively). Perhaps the most striking example in this category is the IGF2BP3 gene (Fig 7).

Reduced expression of the IGF2BP3 gene was reported previously in studies on postnatal development in the mouse [32]. Second, with respect to differences, there is a shift—more evident in the monocyte data (compare Figs 5E, 5F and 6E, 6F)—towards postnatal down-regulation of gene expression that is not explained either by a corresponding net shift in domain size or by a net postnatal increase in H3K27me3-enrichment (see below).

**Fig 8:**
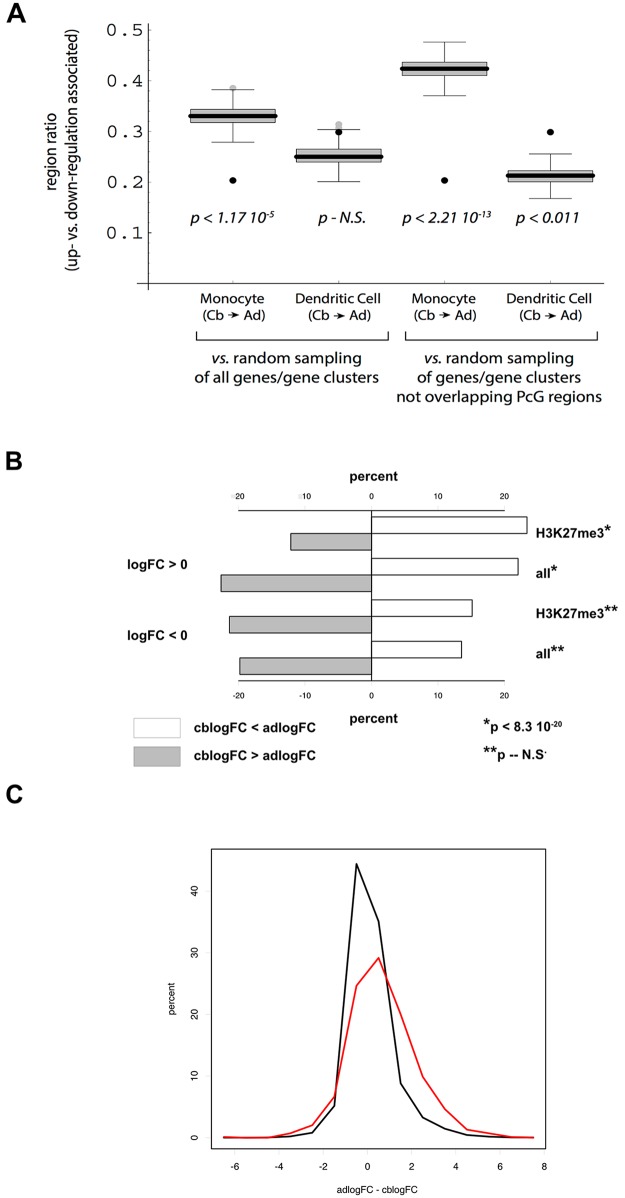
Specificity of PcG-associated gene regulation. A. Ratios of PcG regions linked to up- or down-regulation of gene expression. Region ratios (y-axis) denoted by black points were calculated by dividing: i) the number of regions for which most overlapping genes exhibited postnatal up-regulation, by ii) the number of regions where most overlapping genes exhibited postnatal down-regulation. In the left half of the figure, the box and whisker plots represent distributions of ratio values calculated for sets of ‘virtual’ genome regions with spans corresponding to the experimental PcG region set. Box boundaries and whisker lengths were determined by multiple random sampling, with correction for the level of clustering observed in PcG region-overlapped genes, while p values (Fisher’s test) are based on comparisons of PcG vs. random sampling-derived ratio distributions. The right half of the figure differs from the left only in that random sampling of the genome was filtered to retain only ‘virtual’ regions that did not overlap genes included in the PcG region set. B. Postnatal shift towards increased activation on monocyte to DC differentiation of adult vs. cord blood monocytes. Differentiation-associated log fold change (logFC) designates gene subsets for which both cord blood values (cblogFC) and adult values (adlogFC) were positive. C. Histogram plots of postnatal change in the magnitude of monocyte to DC differentiation-induced activation of gene expression. Black and red lines depict all vs. PcG-associated gene subsets, respectively, with the restriction cblogFC > 0 and adlogFC > 0.

An important aspect of the latter unexplained shift in expression is that it involves a much larger number of genes than that linked to domain size increase. To investigate this in more detail, we focused on the subset of genes characterized by significant postnatal change in monocyte RNA levels (FDR < 0.05). A substantial percentage (28%) of PcG regions defined by the current analysis overlap two or more genes; accordingly, the results shown in Fig 8A (like those in Fig 4) depict ratios of regions overlapping genes where at least the majority of genes for a given region exhibit the indicated direction of change in expression. Further, to address the possibility of differential spatial clustering between PcG-associated and control gene sets, a filter was applied in multiple random sampling tests to normalize for that variable. Comparing ratios of regions encompassing genes with increased vs. decreased RNA levels, it can be seen that in monocytes, but not dendritic cells, there is a dramatic postnatal shift toward reduced gene expression (Fig 8A). For the comparison between PcG-associated and non-overlapping genes, this shift towards down-regulation reaches roughly 2-fold (p < 2.2 10^−13^, Fisher’s test). A much simpler analysis, taking ratios of significant postnatal up- vs. down-regulation in monocytes for genes that either do or do not overlap PcG regions, likewise yields high significance (p < 9.8 10^−19^, Fisher’s test).

It should be emphasized that, while PcG-associated genes are on average strongly repressed (Fig 2), they retain the capacity to respond to differentiation signals; indeed, for all genes significantly up-regulated (FDR < 0.05) during the transition to dendritic cells, the median value is 6.6-fold, whereas the corresponding value is 23.7-fold for PcG-overlapping genes (p < 7.3 10^−83^, Wilcox test). Consistent with the postnatal down-regulation of PcG-associated genes in monocytes, there is a selective bias towards increased activation on monocyte to dendritic cell differentiation in adult- vs. cord blood-derived cells (Fig 8B; p < 8.3 10^−20^, Fisher’s test comparison to all up-regulated genes,). While median postnatal change in the magnitude of differentiation-induced activation is modest (approx. 2-fold) for PcG-associated genes, such change exceeds 4-fold for the top quartile and 14-fold for the top decile; moreover, the overall postnatal change in activation is substantially elevated in the PcG-overlapped subset relative to the non-PcG-restricted control (Fig 8C, p < 2.8 10^−31^, Wilcox test).

### Alternate PcG region parameters

**Fig 9:**
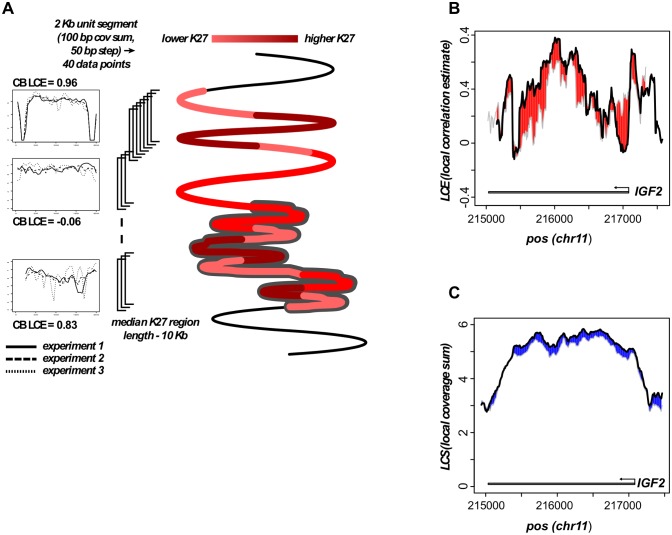
Schematic explanation of local correlation estimate (LCE) parameter. A. Inset panels at left show arbitrarily selected 2 kb segments from cord blood (Cb) monocyte data intended to be representative of the full range of LCE values. B. Example showing young and old adult monocyte LCE region profiles for the IGF2-associated PcG region. C. Example showing young and old adult monocyte local coverage sum (LCS) region profiles for the IGF2-associated PcG region.

The finding that PcG-overlapped genes exhibit distinct postnatal regulation, and that this can be accounted for only in part by PcG region size change, led us to search for new parameters that might link PcG domain topology and gene expression. The algorithms that we introduce here are based in part on the assumption that control loci which may be embedded within PcG regions (and potentially modify local H3K27me3 profiles) can typically be encompassed within a 1–2 kb span. These algorithms were initially adapted from a filter designed to minimize nucleosome-scale noise associated with the most highly variable regions of H3K27me3 enrichment (see Methods). As illustrated schematically in Fig 9, a parameter we term ‘local correlation estimate’ (LCE) was calculated for each of the tiled 2 kb segments that fall within a given PcG region. The descriptor ‘local’ distinguishes this parameter from correlations calculated for an entire PcG region (see below), while the qualifier ‘estimate’ refers to its calculation from the mean value of off-main diagonal pairwise values in a correlation matrix (here a 3 x 3 matrix corresponding to three experiments for each developmental stage). This simple mean-based estimate proved more sensitive than an alternative related to principal component analysis (S4 Fig).

A new measure by which higher order chromatin structures can be compared is shown in Fig 9B. This measure is obtained by calculating the correlation between LCE values (LCE corr) across larger regions. In contrast, a more conventional approach for calculating correlations between region profiles can be based on local coverage sum (LCS) values (Fig 9C). In the IGF2 gene example shown, the LCE- and LCS-based region correlations were found to be 0.64 and 0.99, respectively. The very high latter value suggests that averaging steps, typically used in calculating coverage sums, can lead to considerable loss of information, and that this is potentially related to the masking of heterogeneous epigenome states.

**Fig 10:**
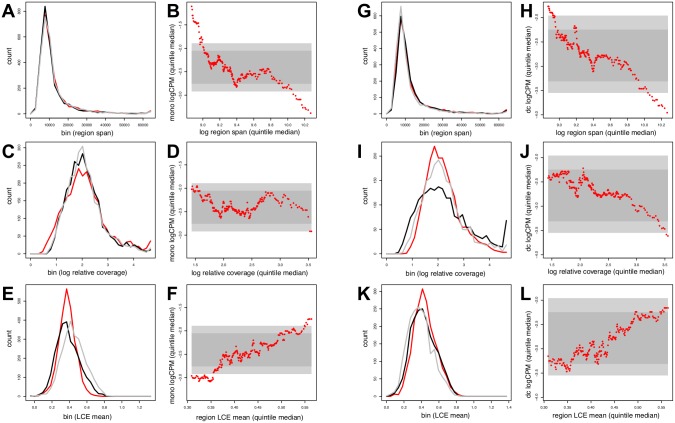
Comparisons between region span-, coverage-, and LCE-based profiling parameters. The left and right halves of the figure show monocyte and DC results, respectively. Panels ACE and GIK, from top to bottom, represent histogram traces that summarize genome-wide region span, coverage, and LCE data. Cord blood (red), young adult (black), and old adult (gray) traces are superimposed. Panels BDF and HJL show quintile-smoothed logCPM expression values as a function of corresponding data types. Rectangles indicate distributions of median logCPM values derived by quintile-scale sampling (above or below gray region, p < 0.05; light gray region, p < 0.005).

The repression of gene expression associated with overlap by large PcG regions, as seen in Fig 2, raised an initial question concerning the regional LCE-based measure, namely, how this would compare to more standard properties such as region span or H3K27me3 enrichment (coverage). Consistent with the focus on structure, we restricted our subsequent analysis to PcG regions with relatively well-conserved boundaries in cord blood monocytes (> = 75% span overlap in three experiments). For reference, region span and region mean coverage (to a lesser degree) are shown to exhibit negative correlations with RNA levels for both monocytes (Fig 10B and 10D) and dendritic cells (Fig 10H and 10J). Somewhat surprisingly, mean region LCE values correlate positively with expression, except over the lowest range (Fig 10F and 10L). This strikingly disparate correlation with respect to RNA levels supports the view that the LCE parameter measures properties of PcG regions that are distinct from span or coverage. Comparison of genome-wide LCE region means in newborn, young and old adult datasets revealed small variations in median values (Fig 10E and 10K), but whether these variations will be reproducible remains to be determined.

### Interrelationships between alternative parameters and gene expression

Of primary interest in this study was whether a topology-based approach, such as described here, could be related to the otherwise unexplained PcG-linked postnatal shift in monocyte gene expression towards down-regulation (Fig 8A vs. the absence of net change in region span seen or coverage as seen in Fig 10A and 10C). For the subset of regions defined by > = 75% span overlap in monocytes, it was found that statistically significant linkage between gene expression change and epigenome structure could be revealed through restricting comparisons by both quantiles and direction of change. To minimize bias due to arbitrary thresholds, quantile cutoffs were evaluated over a series of values in both low and high ranges.

**Fig 11:**
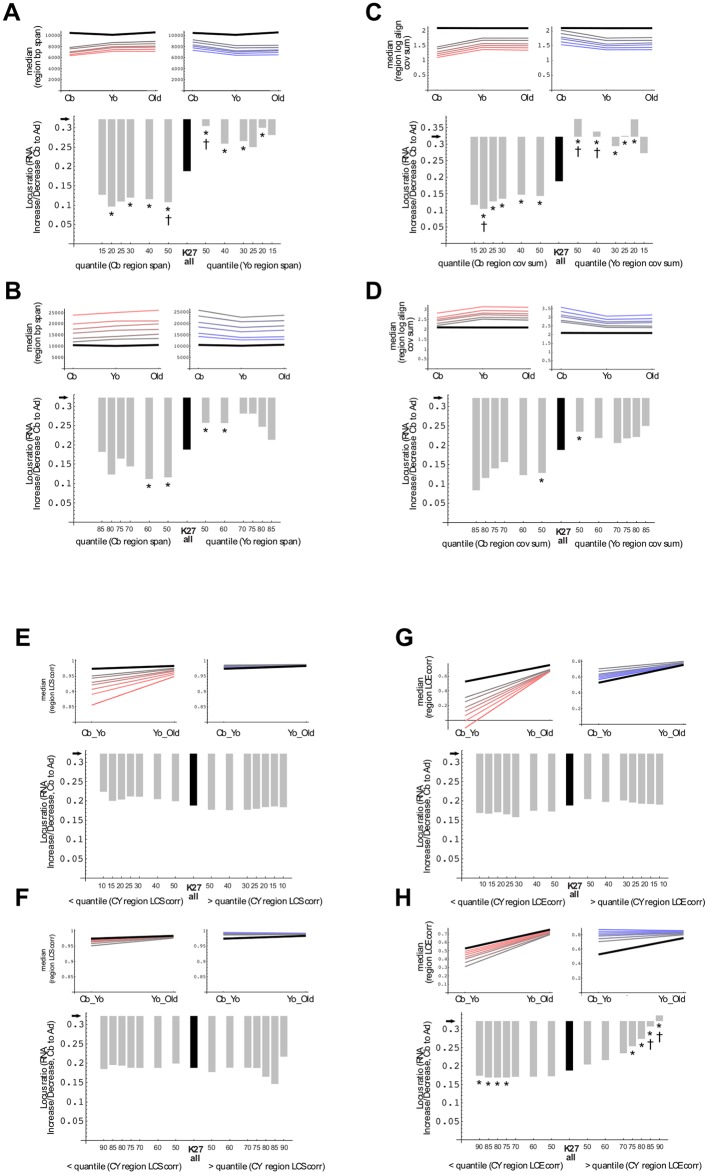
PcG region-linked gene expression change: Conventional vs. alternate parameters. Ratios of significant (FDR < 0.05) up- vs. down-regulated monocyte expression change are plotted, with black arrows indicating ratio for all quantified gene loci and bars depicting deviation. Black bar in each panel shows deviation for all PcG region overlapping loci, whereas for gray bars depict quantile-defined subsets. Plotted in smaller inset panels are total median PcG region values (black line), together with subset median values for the restricted parameter, with shaded colors matching points in the bar graphs. Asterisks denote significant (p < 0.05, Fisher’s test) ratio differences for corresponding left and right subsets. Daggers denote significant change vs. all PcG region-overlapping genes. A. Region span, low range: left, cord blood (Cb) span < specified quantile & Cb < young adult (Yo) span & Cb span < old adult (Ol) span; right, Yo span < quantile & Yo span < Cb span & Ol span < Cb span. B. Region span, high range: left, Yo span > quantile & Cb span < Yo span & Cb span < Ol span; right, Cb span > quantile & Cb span > Yo span & Cb span > Ol span. CD. Subsetting conditions as in AB, but with region coverage sum in place of span parameter. EF. Region LCS (local coverage sum) correlation: left, cord blood-young adult (CY) correlation < specified quantile; right, CY corr > quantile. GH. Region LCE (local correlation estimate) correlation: left, CY corr < quantile; right, CY corr > quantile.

In summarizing the analysis, we again include, for reference, associations between gene expression change and region span or region coverage parameters. Significant correlations were best seen in these reference cases for low range thresholds (Fig 11A, 11B, 11C and 11D).

In contrast, no significant relationships were observed between expression and domain shape (coverage correlation at 2 kb resolution) (Fig 11E and 11F). Crucially, the observed PcG region-associated shift towards expression down-regulation disappeared for high range LCE parameter subsets that are calculated to exhibit minimal change in cord blood-adult comparisons (Fig 11G and 11H; range p < 0.008 to p < 0.035, Fisher’s test). We interpret this last result as supporting a role—in monocytes and perhaps other stem cell-like lineages—for a previously undetected mode of PcG region structure remodeling in postnatal shifts in gene expression.

## Discussion

The analyses presented here were developed in three steps. First, changes in the span of PcG (H3K27me3)-enriched regions were related to altered gene expression associated with differentiation of human monocytes into dendritic cells. This step validated the quality of the data in demonstrating a strong statistical linkage between decreased PcG region span and increased expression of overlapping genes, both for newborn and adult datasets. Second, increases in region span were linked statistically to decreased expression of PcG-associated genes in the context of postnatal development. While based on a cross-sectional design, this second association was found independently for changes in monocyte and dendritic cell RNA levels. Interestingly, in comparisons between newborn and adult monocytes, but not dendritic cells, a widespread shift in expression was observed that could not be explained by net postnatal changes of either PcG region size or enrichment.

The latter finding led us to a third step, namely, introduction of algorithms to quantify PcG domain remodeling that is independent of change in region boundaries. In applying such algorithms, we found that PcG region topology could be linked to both RNA levels and postnatal shifts in monocyte gene expression. The parameter that best reflected this property measures nucleosome-scale conservation (or conversely variegation) of H3K27me3 enrichment profiles within local (kilobase-scale) PcG subregions. By contrast, assessments of overall region shape, based on averaged kilobase-scale coverage sum differences, did not detect comparable postnatal epigenome change.

Whether the epigenome structural features reported here will be reproducible, remains of course to be determined. The number of experiments in the current study used to estimate local conservation/variegation is small; accordingly, a priority for further studies should be calculation of the LCE parameter (or similar parameters) from larger numbers of samples, ideally from large publically accessible epigenome databases. In the meantime, we take as encouraging the finding that the PcG region-specific shift towards postnatal expression down-regulation was not observed for genes overlapped by regions that fell within the highest newborn-young adult LCE correlation quantiles, i.e., regions estimated to have undergone the least remodeling in topology.

In view of the design and size of this study, relatively little emphasis is placed on changes in expression and chromatin architecture at individual loci; nevertheless a few instances seem worthy of comment. Newborn vs. adult comparisons revealed a notable example of expression down-regulation and increased span for a PcG region that overlaps the IGF2BP3 gene promoter (Fig 7). We also observed an 8-fold (FDR < 4.5 10^−9^) development-linked decrease IGF1R RNA levels in association with a 7.5-fold span increase in PcG regions overlapping that gene promoter (S5 Fig). With respect to differentiation, data were presented for the large PcG region overlapping the LMNA locus and its possible differentiation-dependent remodeling, primarily because the LMNA gene is of considerable interest in the contexts of differentiation and aging. The relatively high LMNA RNA levels remain atypical, although it may be noted that such atypical regulation has been described for a subset of heterochromatin-associated genes [33].

With respect to future work, it should be interesting to explore interrelationships between the ChIP-based analyses reported above to postnatal- and age-linked DNA methylation change, as well as to other approaches being actively pursued to characterize higher order chromatin architecture. Genome-wide DNase I footprinting is a well-established method to characterize differentiation- or development-associated variations in chromatin structure [25]. An intriguing question is how shifts in positions of DNase hypersensitive sites relate to strong, discrete transitions in LCE patterns. Likewise, 3C-based approaches are being intensively exploited to probe the structure of large chromatin domains [23, 24], and it should be possible to assess whether LCE-based patterns are sensitive to changes in chromatin domain folding. Super-enhancers are considered to play a fundamental role in control of gene expression, so studies into super-enhancer three dimensional structure and phase separation properties [34] are germane to the results described here. In addition, A/B compartment repositioning of gene control regions during differentiation should be associated with extensive changes in chromatin topology [35].

A very active area of investigation concerns the interaction of PcG complex proteins with RNA [36–38]. Polycomb repressive complex 2 (PRC2) binds RNA promiscuously, but with a significant preference for short guanine tracts which have the potential to form G-quadruplex structures [39, 40]. The functional consequences of PrG protein-RNA interactions are the subject of active debate [40–42]; however, at least in principle, large scale (multi-kilobase) remodeling of PcG ribonucleoprotein architecture, detectable using LCE-based or similar algorithms, may influence the pairing probabilities of distantly spaced control regions such as enhancers to promoters.

The asymmetry in postnatal expression change in monocytes for genes overlapping large PcG regions certainly merits further consideration. Whereas the fraction of such regions spanning up-regulated genes relative to those spanning down-regulated genes is approx. 0.2, the corresponding fraction is greater than 0.4 for randomly selected regions that overlap non-PcG-associated genes/gene clusters with comparable distributions (Fig 8A). One possibility, noted above, is that remodeling of large chromatin domains may redirect enhancer-promoter pairing; if so, the likelihood of reduced expression may be higher than the converse. Another speculative suggestion can be based on the reported strong linkage between PcG region size and packing [26]. Given our observation that region span exhibits a strong negative correlation with the level of expression, the possibility suggests itself that LCE-measurable PcG domain remodeling may be most frequently associated with increased packing density. To test these possibilities, super-resolution microscopy, genome wide Hi-C, and mapping of potential low abundance, cis-acting PrG-bound RNAs, which are powerful yet still resource-intensive approaches, might be prioritized to specific regions on the basis of ChIP-based genome-wide overviews of chromatin topology.

The long-term motivation for the focus here on relatively large chromatin domains is based on the still hypothetical idea that a subset of such domains may function as epigenetic clocks [3–7, 11–13, 43]. While epigenome change through lifespan has been documented repeatedly [9, 10, 12, 13, 15, 16, 22, 44], the question remains whether it plays a causal role in postnatal development or aging. We propose that, if epigenetic structures are to possess sufficiently stable memory to transmit long-term, heritable alterations in gene expression, those structures are most likely to be on the scale characterized in this study. During very early developmental stages, large repressive PcG chromatin domains are established de novo on a widespread basis, but there has been limited success in detecting postnatal epigenome change of a comparable scale, or in linking broad PcG-associated change statistically to gene expression patterns [16, 22, 44]. Our results suggest that, especially in stem cells and stem cell-like lineages, profiling of larger epigenome domains based on local conservation of chromatin topology, a previously unexplored parameter, should add to our understanding of the origins of lifespan progression.

## Methods

### Ethics

All human materials used in this study were received with written informed consent and under approval of the Institutional Review Board of the National Institute of Child Health and Human Development (NICHD), or of the Clinical Center, National Institutes of Health (NIH).

### Human peripheral blood monocyte samples

Cord blood samples were obtained from healthy term newborns. Adult peripheral blood or monocyte-enriched apheresis samples were obtained from healthy volunteers from the NIH Department of Transfusion Medicine.

#### Cell purification and culture

Enrichment for monocytes and differentiation of monocytes to dendritic cells were done as described previously [15, 31]. Cord blood and adult (monocyte-enriched apheresis or whole blood) samples were diluted two- to four-fold in Dulbecco’s PBS (DPBS) with 4% citrate dextrose solution (ACD). Samples were placed on a Ficoll-paque gradient (density 1.077) and centrifuged for 35 min at 900 × g at room temperature. The interphase was transferred to a fresh tube, diluted to 40 ml using DPBS and centrifuged twice for 5 min at 200 × g to reduce platelet contamination. Samples were resuspended in 20 ml of RPMI 1640, 10% fetal calf serum (FCS) and placed on a lower density Ficoll-paque gradient (20 min, 400 × g, room temperature). Interphase cells were collected and washed in DPBS followed by centrifugation for 8 min at 500 × g. Pellets were resuspended in 20 ml PBE (PBS, 0.5% BSA, 2 mM EDTA) and centrifuged for 10 min at 500 × g. Monocytes were further purified by subtractive magnetic cell sorting using a monocyte isolation kit (Miltenyi, Auburn, CA) following the manufacturer’s protocol.

Purified monocytes were washed in DPBS. To obtain dendritic cells (DC), cells were plated in RPMI 1640, 10% FCS, 50 ng/ml GM-CSF, 20 ng/ml IL-4. Cultures were refed every other day and DC were harvested by day 6 or 7. Purities of monocyte and DC preparations were assessed by flow cytometry (FACSCalibur;BD Biosciences, San Jose, CA) with the following antibodies: anti-CD14 FITC, anti-biotin PE (Miltenyi); anti-HLA-DR, and anti-CD86 (BD Biosciences).

### Chromatin immunoprecipitation and ChIP library preparation

Experimental protocols followed those reported earlier [15]. Purified monocytes or in vitro differentiated DCs were crosslinked with 1% formaldehyde solution. Nuclei were isolated and digested with micrococcal nuclease (MN). The soluble chromatin fragments were diluted in ChIP buffer (50 mM Tris–HCl, pH 7.5, 1% NP-40, 0.25% sodium deoxycholate, 150 mM NaCl, 1 mM EDTA, 5 mM Na-butyrate, protein inhibitors) to a final SDS concentration 0.1% and were used for Chromatin Immunoprecipitation (ChIP) using polyclonal anti-trimethylated H3K27 antibody (Millipore #07–449). Sequencing libraries were prepared following Illumina ChIP Sequencing protocol. The DNA was end-repaired and ligated with Illumina sequencing paired end adaptors; after ligation, the DNA was enriched by 18 cycles of PCR with primers complementary to the paired end adaptor sequences followed by agarose gel size selection and purification.

### Genomics analysis

The data discussed in this publication have been deposited in NCBI’s Gene Expression Omnibus [45] and are accessible through GEO Series accession number GSE94631 (https://www.ncbi.nlm.nih.gov/geo/query/acc.cgi?acc=GSE94631).

This study utilized the high-performance computational resources of the HPC Biowulf cluster (http://hpc.nih.gov) at the National Institutes of Health, Bethesda, MD. Bowtie2 alignment of ChIP reads, STAR alignment of RNA-seq reads, and edgeR RNA-seq analysis followed instructions accompanying the respective application packages. C, Perl and R programs for the genomics analyses briefly described below are available on request or via the link https://science.nichd.nih.gov/confluence/display/data/Epigenome+Software+-+Bruce+Howard.

#### Genome annotation

Annotation of all information onto the human genome assembly 37.2 was based on a convention in which chromosomes are represented as zero-initialized binary files. The format of zeros depended on the analysis task: bits for simple 0/1 marks such as inclusion in an enriched region; short ints for ChIP coverage, or C pointers for transcriptome annotation. Use of pointers coupled with ancillary binary and text files in the last instance constituted a sparse array approach to accommodate the varying depth of transcriptome gene overlap. Archiving by tar-gzip compression typically reduced file size by 100–1000 fold.

#### H3K27me3-enriched region boundaries and correlation estimates

Three steps in the algorithm to define enriched region boundaries employed threshold values. The first (coverage minimum) threshold was set on the basis of experimental vs. Poisson enrichment curves (Fig 1 inset). Minor adjustments were allowed based on genome-wide median estimates (range 47–50). A second (composite) threshold specified the fraction (2/3) of experiments in which, at each position, the 100 bp bin coverage minimum was to be met or exceeded. Retained positions were passed to a third (density) threshold which specified that, for 40 consecutive enriched bins, the corresponding span of positions could be no greater than four kilobases, i.e., at least 50 percent of bins scored as positive, given 50 bp steps. With core (2–4 kb) enrichment domains (CEDs) thus defined, the three sets of bin values were converted to log values (or zero if below coverage threshold), and then taken as input to generate corresponding correlation matrices. From correlation matrices, mean correlation estimates were calculated, and CEDs with estimates of zero or less were discarded. Lastly, overlapping CEDs were merged to demarcate region boundaries. The local correlation estimate (LCE) parameter differs from the CED correlation estimate only in being derived from (50 bp) tiled segments of fixed 2 kb length.

#### Region comparisons

To quantify differentiation-associated change in region span (Figs 3 and 4; S2 Fig), region subsets were identified which overlapped at least one gene in common (Build 37.2) and for which span overlap was > = 50% in monocyte or DC alignments. Postnatal change (Figs 5 and 6) likewise required gene and > = 50% span overlap, but in newborn or adult alignments. Outer comparison boundaries were set according the maximum extension of mean boundary-overlapping regions in the six comparison categories, i.e., monocyte (newborn, young, and old) and DC (newborn, young, and old). Fold change assignments were based on region span summation and ratio of the largest regions within the outer boundary field (both criteria satisfied).

## Supporting information

S1 Table Largest H3K27me3-enriched (PcG) regions. Shown is list of top twelve H3K27me3-enriched regions after reverse sort by region span. Gene overlap denotes that any part of transcribed gene region is located within boundaries of the corresponding H3K27me3-enriched region.
(PDF)

S1 Fig IGF2-associated PcG regions in cord blood, young and old monocytes. See Fig 1 and methods for details. Gray bars denote overlapping enriched and non-enriched 100 bp coverage bins.
(PDF)

S2 Fig Inverse relationships between region span and differentiation-dependent adult gene expression change. See Fig 3 for annotation details.
(PDF)

S3 Fig LMNA-associated PcG region: Monocyte vs. dendritic cell (DC) profiles. A. Estimated region boundaries for a large (approx. 50 kb) H3K27me3-enriched domain overlapping the LMNA promoter region in monocyte samples. Blue bar and arrow depict LMNA transcribed region; white rectangle represents region boundaries derived from monocyte data. B. LMNA monocyte and DC expression (red points, log counts per million) relative to all quantified loci (box-whisker plots). Panels CD represent coverage profile comparisons for this region, with differences highlighted in blue.
(PDF)

S4 Fig Mean- vs. eigenvector-derived correlation estimates. Sample of 2000 regions (length 2–4 kb) satisfying enrichment and composite thresholds as described in Methods. meanCE, correlation estimate based on mean of matrix off-main diagonal pairwise values. eigenCE, correlation estimate based on ratio of the first eigenvalue to the trace of the matrix.
(PDF)

S5 Fig IGF1R H3K27me3 regions. PcG (H3K27me3) region spans for monocytes (Mo) and dendritic cells (DC) obtained from cord blood (Cb), young adult (Yo) or old adult (Ol) samples. Blue bar and arrow depict IGF1R transcribed region; white rectangle represents region boundaries derived from adult (Yo and Ol) data.
(PDF)
