# Supplementary material for: Epigenome comparisons reveal linkage between gene expression and postnatal remodeling of chromatin domain topology
Source: PLoS One. 2018 Feb 21;13(2):e0191033. doi: 10.1371/journal.pone.0191033 (PMC5821309; doi:10.1371/journal.pone.0191033)
Supplement: S1 Table — Shown is list of top twelve H3K27me3-enriched regions after reverse sort by region span. Gene overlap denotes that any part of transcribed gene region is located within boundaries of the corresponding H3K27me3-enriched region. (PDF) [file pone.0191033.s001.pdf]

chr	*start*	*span*	*gene overlap*
chr12	54293601	178133	HOXC13 HOXC12 HOTAIR HOXC11 HOXC10 MIR196A2 HOXC9 HOXC8 HOXC4 HOXC5 HOXC6 MIR615 FLJ12825 LOC100240735
chr13	99648001	176233	DOCK9
chr2	176915101	148050	EVX2 HOXD13 HOXD12 HOXD11 HOXD10 HOXD9 HOXD8 LOC100506783 MIR10B HOXD4 HOXD3 LOC401022 LOC100506798 HOXD1
chr7	27181851	120700	LOC100133311 HOXA5 HOXA6 HOXA7 HOXA9 MIR196B HOXA10 HOXA11 HOXA11AS HOXA13 NCRNA00213 LOC100129463 EVX1
chr5	140703251	119166	PCDHGA1 PCDHGA2 PCDHGA3 PCDHGB1 PCDHGA4 PCDHGB2 PCDHGA5 PCDHGB3 PCDHGA6 PCDHGA7 PCDHGB4 PCDHGA8 PCDHGB5 PCDHGA9 PCDHGB6 PCDHGA10 PCDHGB7 PCDHGA11 PCDHGB8P PCDHGA12 PCDHGB9P
chr1	202580001	110350	SYT2
chr17	46639051	101000	HOXB2 LOC100506360 HOXB3 HOXB4 MIR10A hCG_2042068 HOXB5 HOXB6 HOXB7 HOXB8 HOXB9 MIR196A1
chr10	102831551	97233	TLX1NB TLX1 RNY5P7
chr19	32802551	93500	ZNF507
chr5	38421301	84500	EGFLAM LIFR
chr1	149932551	84300	OTUD7B TRNAA16
chr3	147066851	81766	ZIC4 ZIC1 FLJ30375

S1 Table. List of largest H3K27me3 regions
