# Supplementary figures and images for: Epigenome comparisons reveal linkage between gene expression and postnatal remodeling of chromatin domain topology
Source: PLoS One. 2018 Feb 21;13(2):e0191033. doi: 10.1371/journal.pone.0191033 (PMC5821309; doi:10.1371/journal.pone.0191033)

S1 Fig.

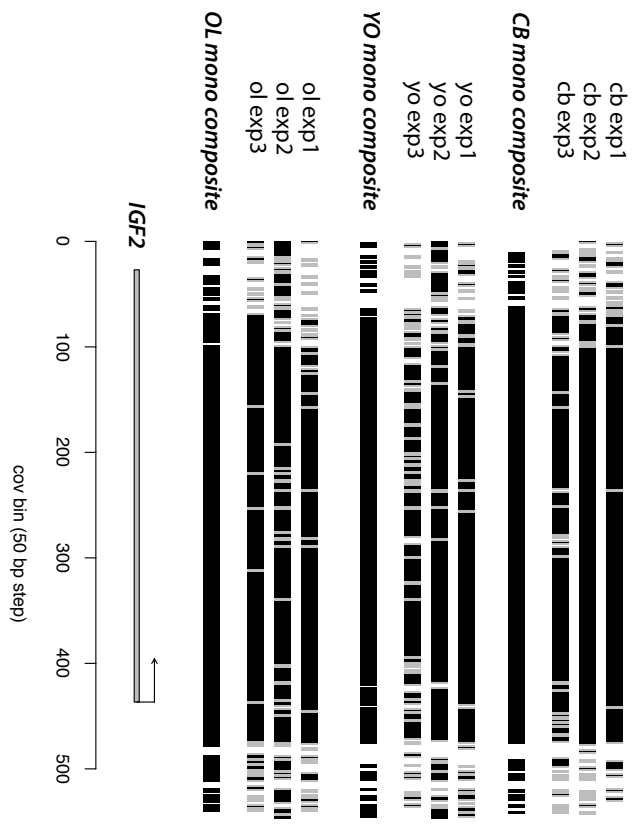

Supplement: S1 Fig — See Fig 1 and methods for details. Gray bars denote overlapping enriched and non-enriched 100 bp coverage bins. (PDF) [file pone.0191033.s002.pdf]

S2 Fig.

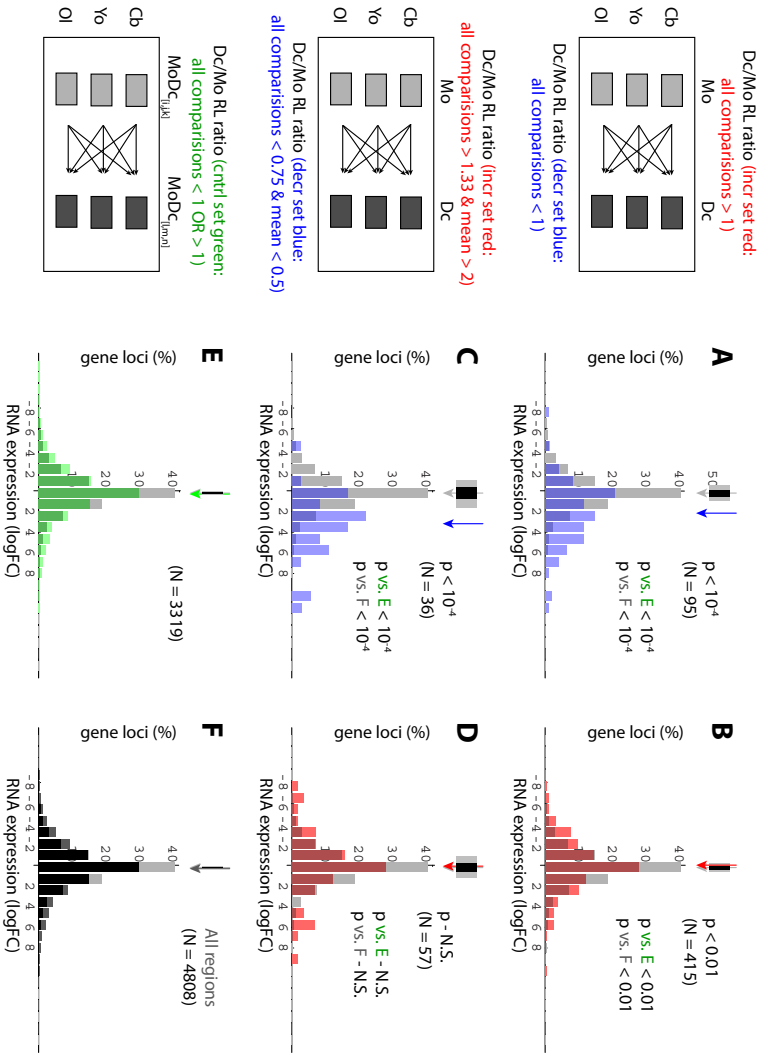

Supplement: S2 Fig — See Fig 3 for annotation details. (PDF) [file pone.0191033.s003.pdf]

S3 Fig.

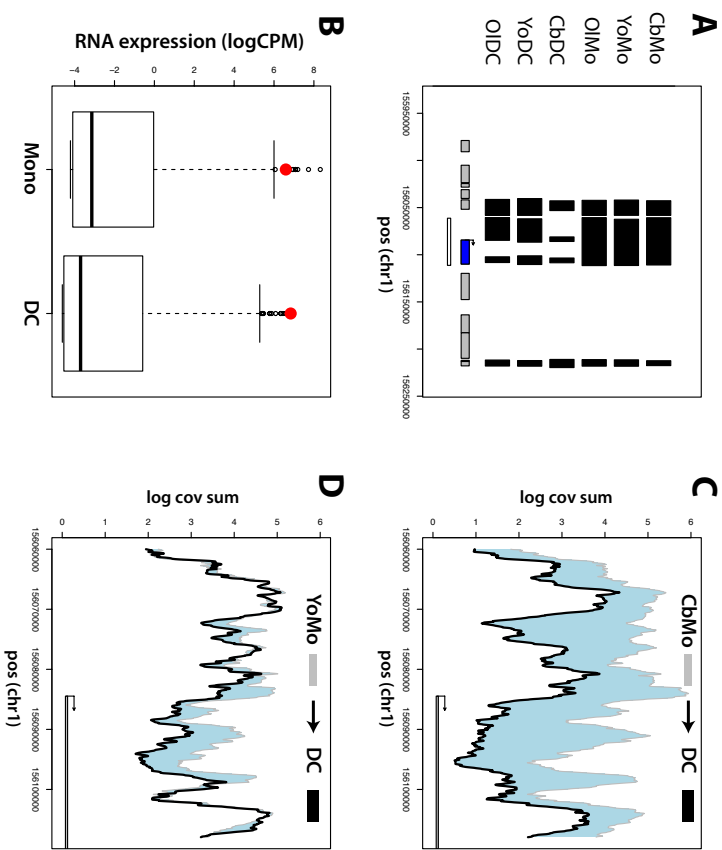

Supplement: S3 Fig — A. Estimated region boundaries for a large (approx. 50 kb) H3K27me3-enriched domain overlapping the LMNA promoter region in monocyte samples. Blue bar and arrow depict LMNA transcribed region; white rectangle represents region boundaries derived from monocyte data. B. LMNA monocyte and DC expression (red points, log counts per million) relative to all quantified loci (box-whisker plots). Panels CD represent coverage profile comparisons for this region, with differences highlighted in blue. (PDF) [file pone.0191033.s004.pdf]

S4 Fig.

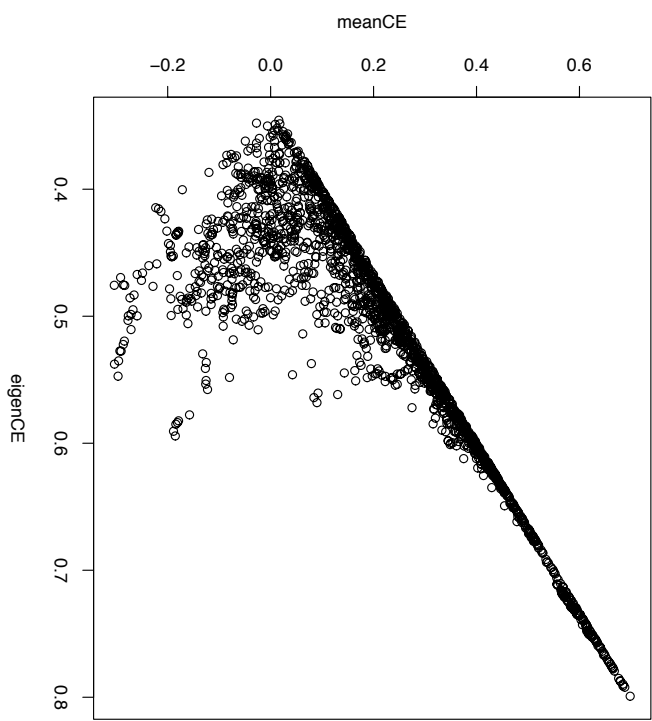

Supplement: S4 Fig — Sample of 2000 regions (length 2–4 kb) satisfying enrichment and composite thresholds as described in Methods. meanCE, correlation estimate based on mean of matrix off-main diagonal pairwise values. eigenCE, correlation estimate based on ratio of the first eigenvalue to the trace of the matrix. (PDF) [file pone.0191033.s005.pdf]

S5 Fig.

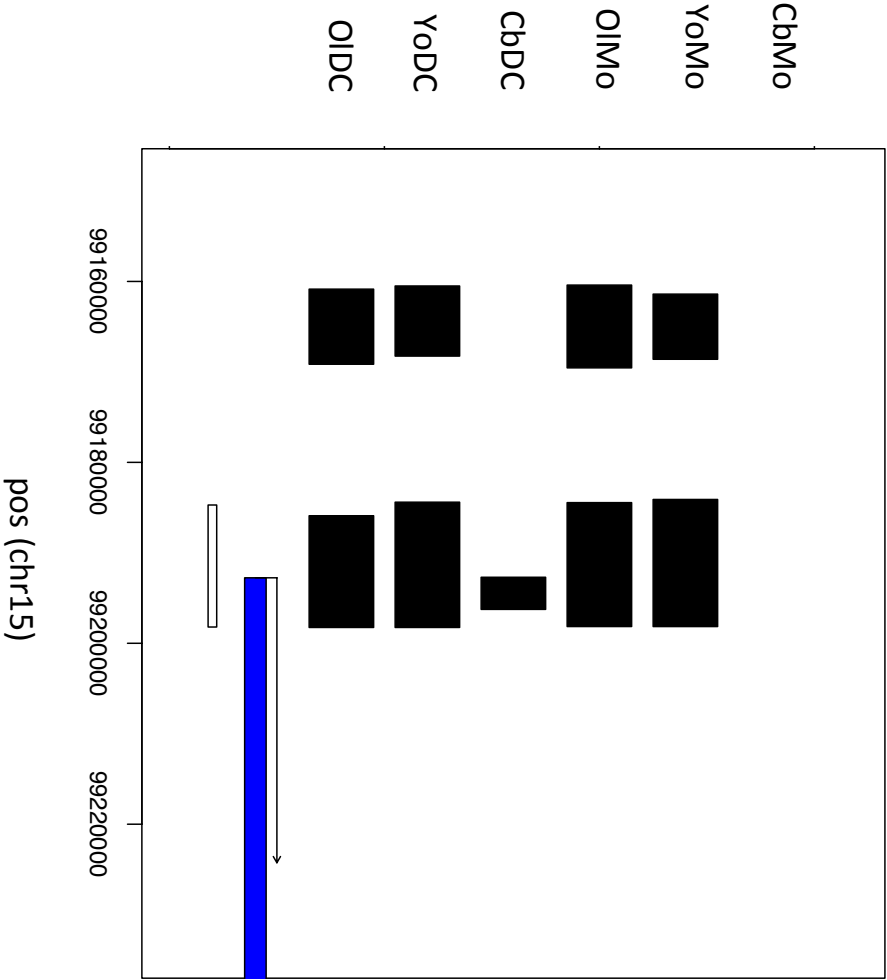

Supplement: S5 Fig — PcG (H3K27me3) region spans for monocytes (Mo) and dendritic cells (DC) obtained from cord blood (Cb), young adult (Yo) or old adult (Ol) samples. Blue bar and arrow depict IGF1R transcribed region; white rectangle represents region boundaries derived from adult (Yo and Ol) data. (PDF) [file pone.0191033.s006.pdf]
